# Substitution of the quantitative serological component in the 2010 criteria for RA with qualitative presence of three autoantibodies yields similar performance: response to the article by Regueiro et al.

**DOI:** 10.1186/s13075-020-02182-3

**Published:** 2020-04-15

**Authors:** Bastiaan T. van Dijk, Leendert A. Trouw, Annette H. M. van der Helm-van Mil, Tom W. J. Huizinga

**Affiliations:** 1 Department of Rheumatology, Leiden University Medical Centre, P.O. Box 9600, 2300 RC Leiden, the Netherlands; 2 Department of Immunohematology and Blood Transfusion, Leiden University Medical Centre, Leiden, the Netherlands; 3 Department of Rheumatology, Erasmus Medical Centre, Rotterdam, the Netherlands

Dear Editor,

Classification criteria are meant for research. Validation of tests that measure individual components of criteria is crucial for the primary goal of classification criteria, namely that similar patients are included in scientific research wherever in the world. Since the 2010 criteria for RA were introduced, there have been concerns that sending the same serum sample to different laboratories often yields different levels [1], making the adjudication of points to levels problematic. An alternative to levels is the yes/no presence of autoantibodies which is supposed to be more reliable between different laboratories. Therefore, the recent report by Regueiro et al. is relevant. They reported that qualitative testing of three RA autoantibodies (including anti-CarP) demonstrated similar or slightly better test results than the 2010 ACR/EULAR serological criteria that incorporate quantitative results of ACPA and RF testing [2].

To test whether the results of Regueiro et al. can be replicated, we investigated the baseline serum samples of patients from the Leiden Early Arthritis Clinic (Leiden-EAC) from 1993 to February 2015. For the current analyses, we included patients newly presenting clinically apparent arthritis with a clinical suspicion of RA or undifferentiated arthritis (UA) at baseline, regardless of fulfilment of classification criteria. Patients who at baseline received an arthritis diagnosis other than RA or UA were excluded. The Leiden-EAC is a Dutch inception cohort including patients with clinical arthritis with a symptom duration < 2 years at presentation, which has been described previously [3]. The presence of RF, ACPA and anti-CarP was determined as described previously; for anti-CarP, it concerned an in-house ELISA [4]. We compared test characteristics and odds ratios (ORs) between the ACR/EULAR 2010 criteria, incorporating quantitative results (levels) of ACPA and RF, and the modified criteria using qualitative results (presence) of anti-CarP, RF and ACPA (5 points for 3; 3 for 2; 1 for 1 concordant antibody/-ies, as proposed by Regueiro et al.) while maintaining ≥ 6 points as the cut-off. Fulfilling the 1987 criteria after 1 year was used as the gold standard for RA.

Of 2429 consecutive patients with a clinical suspicion of RA or UA, 2010 had data on all three antibodies. Of these, 2000 had 1-year follow-up data and were studied. Test characteristics were found to be similar between the ACR/EULAR 2010 and modified criteria (Table 1). Most importantly, sensitivities were 84.5% (95% CI 82.4–86.7) and 82.3% (80.0–84.7), and specificities were 68.8% (65.9–71.7) and 71.6 (68.8–74.5), respectively.

**Table 1:** Performance of the modified 2010 criteria (incorporating the concordance serological score proposed by Regueiro et al.) compared to the original ACR/EULAR 2010 criteria for RA, with RA according to the 1987 criteria at 1 year as the gold standard

Serological component	Sensitivity, % (95%CI)	Specificity, % (95%CI)	PPV, % (95%CI)	NPV, % (95%CI)	OR (95%CI)
2010 ACR/EULAR (quantitative)	84.5 (82.4–86.7)	68.8 (65.9–71.7)	74.9 (72.4–77.4)	80.2 (77.4–82.9)	12.1 (9.7–15.0)
Modified (qualitative)	82.3 (80.0–84.7)	71.6 (68.8–74.5)	76.2 (73.7–78.7)	78.7 (75.9–81.4)	11.8 (9.5–14.6)

*PPV* positive predictive value, *NPV* negative predictive value, *OR* odds ratio, *CI* confidence interval

In conclusion, we replicated the findings from Regueiro et al. and observed that methodology based on qualitative testing of anti-CarP, RF and ACPA yields similar test characteristics as the original methodology based on quantitative testing of RF and ACPA. Assuming that the results will be similar when commercially available anti-CarP tests are done, and because auto-antibody levels are more difficult to harmonise between different laboratories [1], these results suggest that quantitative testing can possibly be replaced by qualitative testing.

## Data Availability

Data are available from the corresponding author on reasonable request.
